# Developing Computational Model to Predict Protein-Protein Interaction Sites Based on the XGBoost Algorithm

**DOI:** 10.3390/ijms21072274

**Published:** 2020-03-25

**Authors:** Aijun Deng, Huan Zhang, Wenyan Wang, Jun Zhang, Dingdong Fan, Peng Chen, Bing Wang

**Affiliations:** 1 Key Laboratory of Metallurgical Emission Reduction & Resources Recycling (Anhui University of Technology), Ministry of Education, Ma’anshan 243002, China; ajdeng@163.com; 2 School of Metallurgical Engineering, Anhui University of Technology, Ma’anshan 243032, China; 3 Department of Engineering, University of Leicester, Leicester LE1 7RH, UK; 4 School of Electrical and Information Engineering, Anhui University of Technology, Ma’anshan 243032, China; 1135130589@163.com (H.Z.); 1743388711@163.com (W.W.); 5 Co-Innovation Center for Information Supply & Assurance Technology, Anhui University, Hefei 230032, China; wwwzhangjun@ahu.edu.cn

**Keywords:** protein interaction sites, unbalanced data sets, overlapping regions, XGBoost

## Abstract

The study of protein-protein interaction is of great biological significance, and the prediction of protein-protein interaction sites can promote the understanding of cell biological activity and will be helpful for drug development. However, uneven distribution between interaction and non-interaction sites is common because only a small number of protein interactions have been confirmed by experimental techniques, which greatly affects the predictive capability of computational methods. In this work, two imbalanced data processing strategies based on XGBoost algorithm were proposed to re-balance the original dataset from inherent relationship between positive and negative samples for the prediction of protein-protein interaction sites. Herein, a feature extraction method was applied to represent the protein interaction sites based on evolutionary conservatism of proteins, and the influence of overlapping regions of positive and negative samples was considered in prediction performance. Our method showed good prediction performance, such as prediction accuracy of 0.807 and MCC of 0.614, on an original dataset with 10,455 surface residues but only 2297 interface residues. Experimental results demonstrated the effectiveness of our XGBoost-based method.

## 1. Introduction

Protein-protein interaction (PPI) is the main way to realize the regulation of biological information, and it is an important factor to determine the fate of cells [1,2,3]. The study of protein-protein interactions is the basis for understanding life activities and one of the most important topics in the post-genome era. With the completion of the human genome project, the data in the protein sequence database has increased dramatically, and the number of protein structures and PPIs are much in arrear of that of sequences. Identification of protein-protein interaction sites (PPIS) by experimental methods is not only time-consuming and laborious, but also suffers from high false positives and negatives.

Fortunately, predicting protein-protein interaction sites using computational methods has become a hot topic with the development of machine learning algorithms [4,5,6,7,8]. Previous studies showed that support vector machine (SVM) and its improved methods can predict effectively protein interaction sites [9,10,11,12,13,14]. Computational algorithms such as random forests, KNN, and Naive Bayes Classifier have been also applied to the prediction of PPIs [15,16,17,18]. Wang et al. proposed a new method for predicting protein interaction sites in hetero-complexes using a radial basis function neural network (RBFNN) set model, which uses only evolutionary conservation information and spatial sequence profile of proteins, and achieved a good predictive result [19].

However, only a small number of protein interaction sites were experimentally validated within current databases, which causes a highly imbalanced distribution of interaction or non-interaction sites, and therefore decreases the predictive performance of computational models in predicting protein functional sites. There are some works that tried to address the problem of sample imbalance. Li proposed RBFIS (improved radial basis function by SMOTE) to solve the problem of sample imbalance [20]. The down-sampling methods based on easy ensemble and balance cascade were proposed to solve the problem of unbalanced data [21]. Wang et al. proposed a manifold learning approach, and tried to re-balance the original dataset using clustering algorithm [22]. Although these methods have made great progress in predicting protein interaction sites, the random down-sampling they adopted may result in the missing of some important sample information, and the risk of overfitting in the prediction of protein interaction sites.

In this work, two imbalanced data processing strategies based on XGBoost algorithm were proposed to re-balance the original dataset from inherent relationship between positive and negative samples in current dataset, which can effectively reduce the influence of data imbalance problem, and therefore improve the prediction performance of protein interaction sites. Our work focuses on the extraction of related feature attributes for negative samples selection, and applies XGBoost algorithm to improve the prediction performance of protein interaction sites, which is a very effective machine learning algorithm can handle large-scale data efficiently [23]. The main contributions of this study are to extract the characteristics of amino acids, which can reduce the data dimension and increase the speed of operation, and propose two down-sampling methods, i.e., repetitive nearest neighbor rule (RENN) and instance hardness threshold (IHT), to select non-interface residues with high reliability. Experimental results showed that our proposed method achieved a good prediction performance, such as prediction accuracy of 80.7% and sensitivity of 81.2%.

## 2. Results

The goal of this study is to identify interaction sites on the surface of proteins and build predictors using a computational method. The data set used herein is the 91 non-redundant protein chains which have a total of 10,455 surface residues; 2297 of them are interfacial residues and 8158 non-interface residues. The work builds a balanced data set from the original unbalance one to improve the prediction performance of protein interaction sites from protein sequences.

### 2.1. Evaluation Criteria

Traditional machine learning algorithms usually use accuracy as an evaluation index. However, for an imbalanced dataset, the evaluation of classifiers tended to reduce the classification effect of a few types of samples. Therefore, multiple evaluation indicators, such as accuracy (Acc), sensitivity (Sen), precision (Pre) and specificity (Spe), are used to evaluate the prediction results in this study. As an effective indicator for the classification performance of unbalanced data sets, F−measure is a weighted harmonic mean of precision and sensitivity. Matthews correlation coefficient (MCC) describes the degree of correlation between the predicted and actual residue classification, and its value ranges from −1 to 1, where −1 indicates the worst prediction, and 1 the best prediction.

(1) $$Acc=\frac{TP+TN}{TP+FP+TN+FN}$$

(2) $$Sen=\frac{TP}{TP+FN}$$

(3) $$Pre=\frac{TP}{TP+FP}$$

(4) $$Spe=\frac{TN}{FP+TN}$$

(5) $$F-measure=2\times \frac{Pre\times Sen}{Pre+Sen}$$

(6) $$\begin{array}{l} MCC=\frac{\left(TP\times TN-FP\times FN\right)}{\sqrt{\left(TP+FP)\times (TP+FN)\times (TN+FP)\times (TN+FN\right)}} \end{array}$$

where TP represents the number of interfacial residues that are actually interfacial residues, FP represents the number of interfacial residues that are actually non-interface residues. TN represents the number of non-interface residues that are predicted to be non-interface residues, and FN indicates the number of non-interface residues that are actually interfacial residues.

### 2.2. Predictive Performance in Two Balanced Modes

Although the purpose of RENN and IHT is same, the balance strategy is different. RENN will repeatedly remove the noise of non-interface residues and overlapping areas of samples until it cannot be removed. IHT can only remove non-interface residues and achieve a balance with interface residues. The number of samples of data processed by two sampling methods is shown in Table 1.

Table 2 shows the performance of IHT and RENN in predicting protein-protein interaction sites. It can be seen that both methods can effectively predict protein-protein interaction sites, i.e., 0.707 of accuracy for RENN and 0.807 for IHT when they are combined with XGBoost predictor. It can be found that a relatively high MCC values for both predictors show the effectiveness of the features extracted from evolutionary conservation in differential interface residues from non-interface ones. With Comparison to RENN, IHT shows a better prediction, which can achieve 9.94% in accuracy, 4.66% in sensitivity and 7.69% in F-score in the performance measures.

From the experimental results, it can be seen that our hypothesis there are a large number of sample overlap areas within the data set is correct. Although both of RENN and IHT can re-balance the original dataset, IHT is more efficient in processing sample overlap areas, which may be the main reason a better prediction performance can be achieved based on a similar number of positives and negatives. According to specificity and sensitivity, IHT-XGB can improve the recognition rate of a few samples when that of most samples does not decrease.

### 2.3. Comparison of Unbalanced and Balanced Data Sets

**Figure 1:**
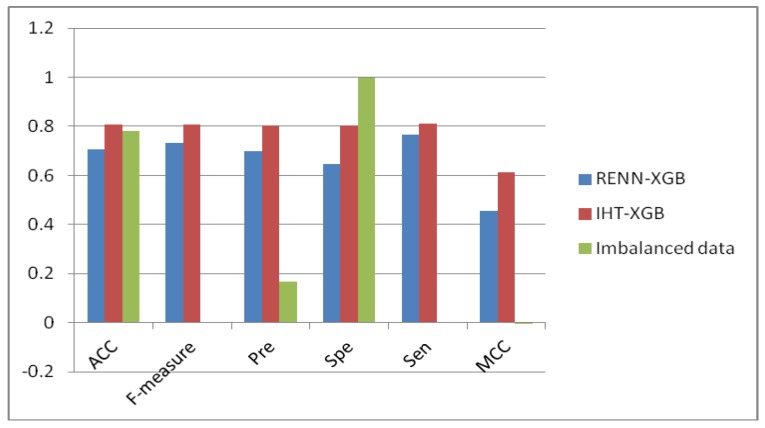
Performance comparison between the original and re-balanced datasets.

To infer the effect of dataset balance, we compare the results by the same XGBoost predictor on the original unbalanced dataset and balanced dataset using RENN and IHT methods. Experimental results show that when the data sets are unbalanced, the accuracy of the model reaches 0.78, but the sensitivity is only 0.0021, and the F value is 0.0042. The very low value of sensitivity shows that only a very small number of positives can be identified, and most of predictions are negative, which means that prediction results tend to the powerful categories within an imbalanced dataset, especially in the original dataset in this work where the negative samples far exceed the positive samples. The highest specificity on the original imbalanced dataset, such as almost equal to 1, shows almost all of negatives can be predicted, which further demonstrated the overfitting problem for unbalanced data processing. The comparison of prediction performance on the original unbalanced and re-balanced dataset can be found in Figure 1.

Detailed information about the predictions on the balanced dataset based on the two down-sampling methods proposed in this work and the original dataset can be seen in Table 3. It can be found that the number of negative samples is much larger than that of positive samples, and the number of TN and FN is much larger than that of TP and FP. After classification, the number of negative samples is 346 times that of positive samples. For the balanced data set after sample sampling, the proportion of negative samples and positive samples after classification is equivalent, and the prediction effect is far better than that of unbalanced data set.

## 3. Discussion

### 3.1. Comparison with Other Methods

**Figure 2:**
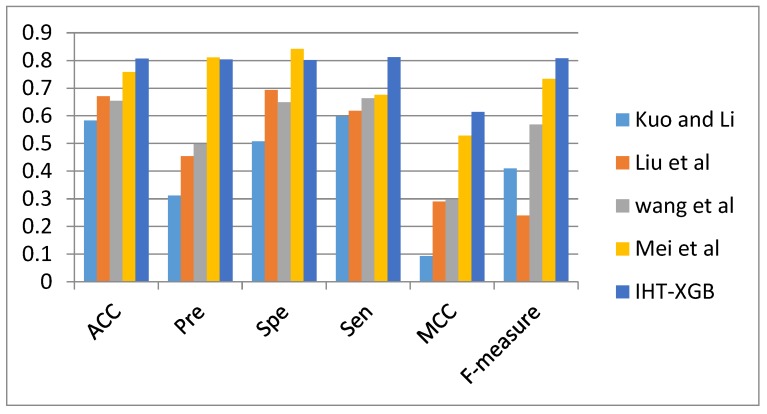
Prediction performance comparison of IHT-XGB method with four previous works.

To analyze the performance of our proposed methods, we compared the prediction results with other approaches in protein interaction sites identification. To make the comparison more convincing, this work compared the prediction performance of the presented re-balanced strategies with four previous studies using the same dataset. Wang’s work extracted the residue evolutionary conservation and sequence profile to infer protein interaction sites, which is already a benchmark method for the prediction performance comparison [24]. Kuo and Li developed an SVM model to predict protein-protein interaction sites by extracting five different sequence features [25]. Liu et al. identified protein-protein interaction sites with temperature factor, sequence profile and accessible surface area [26]. Mei et al. tried to predict protein-protein interaction sites by using semi-supervised SVMs [27]. All of these four methods randomly selected subset of non-interacting sites in the prediction. The performance comparison of five works is shown in Figure 2. 

It can be seen from Figure 2 that the proposed IHT-XGB method can achieve a better overall performance than the other four approaches can do. For all of the methods, IHT-XGB is not only as good as Mei’s work on the measures of *Pre* and *Spe*, and can outperform all of the other four measures. The accuracy of IHT-XGB is 80.71%, which is 4.9% higher than that of Mei’s, 15.3% than Wang’s, 13.6% than Liu’s, and 22.4% than Li and Kuo’s work. The MCC of IHT-XGB is 0.614 indicates that the features used in predictors can distinguish interaction and non-interaction residues effectively. The high *Sen* of 0.812 shows that IHT-XGB can identify more protein interface residues from protein chains. Furthermore, it can be found that the value of IHT-XGB on the five measures, i.e., ACC, Sen, Spe, Pre, and F-measure are very close, which indicated that the accurate prediction rate in positives and negatives are also close, and there is no tendency for the predictor to overfitting to the majority class. 

In this work, the features of residues used herein are similar to that in Kou and Li’s work, but the performance are totally different, and the comparison results shows that data re-balance is necessary and effective. In addition, the experimental results also demonstrated our hypothesis that there are a large number of overlapping regions between the positives and negatives in the original dataset. If the overlapping regions of samples were reasonably processed, such as in the case of IHT-XGB, the prediction performance of the model can be improved dramatically.

### 3.2. Prediction Performance in Independent Benchmark Datasets

To further evaluate the prediction performance of IHT-XGB model, three widely used datasets are used for independent testing. All of them are publicly available from previous studies: Dset_186, Dset_72 and Dset_164 [28,29,30]. These datasets consist of 186, 72, and 164 protein sequences, where the number of interaction sites is 1923, 5517, and 6069 within them, respectively. All of the protein sequences are extracted from PDB database with following the same rules, such as less than 25% sequence homology identity, less than 3.0 Å of the resolution in X-ray crystallography, and removing transmembrane proteins. The prediction performance of the proposed model is shown in Table 4. It can be found that the values of six measures achieved in these three independent benchmark datasets are lower than that in the dataset used in this work, but it is reasonable because the IHT-XGB model is built on this original dataset. 

Table 4 also shows the prediction performance of five models, i.e., SSWRF, LORIS, PSIVER, SCRIBER, and DELPHI, in identification of protein-protein interaction sites [31,32,33,34,35,36,37]. The highest results in each performance measures for different models are highlighted as bold type. It can be seen that the IHT_XGB model can achieve the highest values in F-measure, Pre and MCC in all of three datasets, and the best Sen in Dset_72 and Dset_164. The proposed model also achieves the second highest performance of *Spe* in three datasets and the second Sen in Dset_186. Although DELPHI can get the best Acc and Spe, the other measures are obviously lower than what IHT_XGB can reach. The *Sen* values are around 0.27−0.35 in all three benchmark datasets means DELPHI can predict only a small number of protein interaction sites correctly. It can also be found that the IHT_XGB model can get a balanced performance among the different measures, which is similar with it did in the dataset used in this work. The results demonstrated the effectiveness of the proposed model.

### 3.3. Visualization of Experimental Results

**Figure 3:**
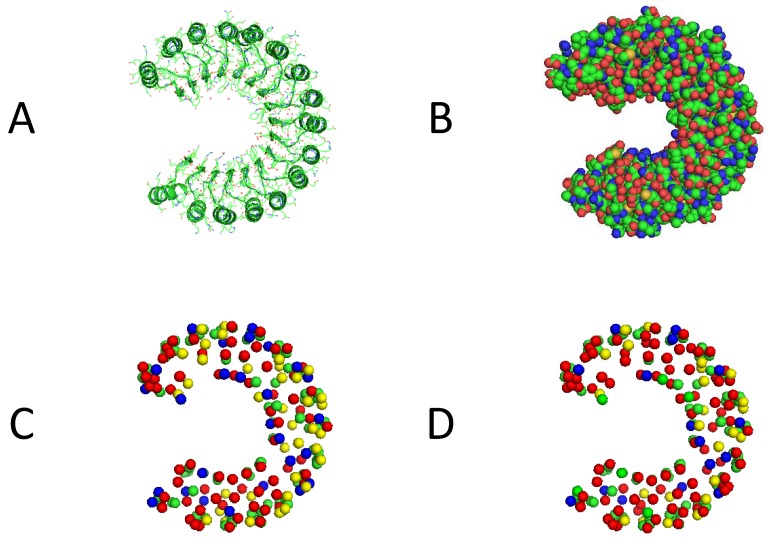
Visualization results of predictions by the proposed methods. (**A**), (**B**) represent the cartoon and spheres form of 1a4y-a, and (**C**), (**D**) represent predictions based on RENN-XGB and IHT-XGB methods, where green, red, yellow, and blue ball represent the predictions of TP, TN, FP and FN, respectively.

To show the results of the proposed methods, a molecular visualization tool, pymol, is adopted to demonstrate our predictions. Figure 3 shows the cartoon and spheres forms of protein chain 1a4y-a and the results of RENN-XGB and IHT-XGB prediction methods. It can be found that by predicting the surface residues involved, our method can improve the overall prediction performance, and successfully predict most interfacial and non-interfacial residues. It can also be seen that IHT-XGB can get better predictions than RENN-XGB.

### 3.4. Limits of Prediction Validation

In this work, the experimental results show that the proposed method can identify interaction sites from protein sequence and outperform other computational algorithms in prediction accuracy. However, those predictions only provide potential options for experimental validation, which can dramatically decrease the number of site candidates, and enhance pertinence of experimental design. Actually, identifying potential interacting sites is only the first step toward understanding the impact of a protein interaction, and the interaction occurs or not is dependent on the binding kinetics of the interaction as well as the environment of the interactions within a cell. 

The association rates often play a critical role in the formation of protein-protein, which is determined by the free energy difference of the bonded and non-bonded states, and it is also the fundamental theory of protein docking [38]. Theoretically, the strength of a protein-protein interaction can be characterized by a dissociation constant $K_{D}=k_{d}/k_{a}$, where $k_{d}$ is the dissociation rate constant and $k_{a}$ is the association rate constant. Many commonly used techniques provide measurements of $K_{D}$, which can be calculated by the concentration of free proteins, but most of them do not offer the real-time measurements of $k_{d}$ and $k_{a}$ [34]. 

In future work, the prediction results in this work will be validated by protein-protein docking method. Although the goal of docking is the prediction of three-dimensional structure of the protein complexes using computational modeling methods, the docking areas can provide an important clue for evaluating the accuracy of our prediction. Obviously, if the predicted interaction sites in this work locate onto the interfaces between proteins, they contribute the formation of protein-protein interaction, and therefore have high possibility to be true positive sites. 

## 4. Materials and Methods

### 4.1. Dataset

Currently, there is no uniform standard dataset for prediction of protein interaction sites due to the lack of corresponding selection criteria when selecting data sets. Therefore, to compare with other research methods, the dataset of this study was screened from the dataset containing 170 transient protein interactions used by Ansari and Helms, which is a gold dataset widely used in protein studies [39]. To ensure the quality of the experimental data, we remove the antibody-antigen interaction, and delete the protein chain pairs with less than 50 residues, leaving only the chain with most interface residues. In addition, the BLASTCLUST program was used to remove proteins with sequence similarity greater than or equal to 30%. Finally, only 91 non-redundant protein chains were left in our work.

The definition of residues is the same as that many previous works adopted [6,40]. The relative accessible surface area (RASA) of amino acid residues with maximum accessible surface area of more than 16% is defined as surface residues. Of them, the distance between two residue carbon atoms in the interaction chain greater than 1.2 nm are defined as non-interface residues, and conversely, defined as interfacial residues. Finally, 91 protein chains in this work produced 10,455 surface residues, of which 2297 were interface residues and 8158 were non-interface residues. Obviously, the data set in an unbalanced data set.

### 4.2. Feature Extraction

In this work, each protein residue is represented by evolutionary conservation scores. A total of five features were extracted based on the evolutionary conservative type of amino acids, such as four features from HSSP database, i.e., residue space sequence, sequence information entropy, relative entropy, and residue sequence weight, as well as one feature, i.e., residue conservative fraction, extracted from Consurf Serve [41].

To take the synergies of neighborhood residues in protein chain, the residue-centered sliding window with 11 lengths and its 10 nearest neighbors on the protein surface are used to encode the residue’s eigenvectors. These 10 residues can be processed at the local interface around the target residue, and each of them, just like the target residue, is vectorized into a 24-dimensional feature. Finally, 264-dimensional vectors of each residue are obtained and used to construct future predictors.

### 4.3. Unbalanced Data SETS Processing

In our work, the proportion of positive samples in our dataset is only 21.9% of the total samples. This imbalance is very common in the studies of protein interaction sites. If a predictive classifier is constructed based on this dataset directly, the model will tend to negative samples, which will lead to inaccurate predictions of positive samples.

**Figure 4:**
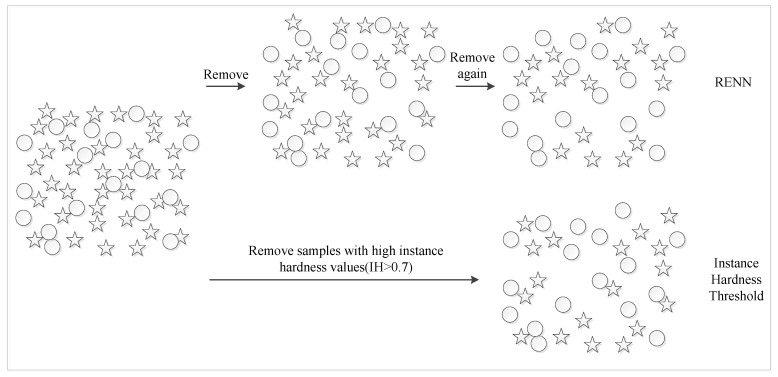
RENN and IHT algorithm schematic. Herein, the circles denote the positive samples, and the stars are negative ones.

From the definitions of the surface and interface on protein chains, there should be some false positives and negatives existing within the original dataset. It can be found that the original dataset was determined by the RASA and the distance of carbon atoms after the protein complexes formed from single chains, which is a hard threshold and cannot totally describe the functional difference between interface and non-interface residues. The number of negative samples is obviously larger than that of positive samples, and there must be a lot of negative samples between positive samples. The hypothesis of our work is that there are a large number of sample overlap areas within the data set, and deduction of the impact of sample overlap can improve the quality of the original dataset, and therefore is of significant for the prediction of protein interaction sites. In this paper, two sample sampling techniques are provided to effectively deal with overlap problems between different categories’ protein residues (Figure 4).

#### 4.3.1. Instance Hardness Threshold

Instance hardness (IH) was proposed by Michael R. Smith, which can effectively deal with the class overlap problem within data [42]. This method adopted the concept of IH property to represent the probability that the data points are misclassified in the training set. An edge between two or more classes or a data sample with noise characteristics has a higher IH value because the learning algorithm forces them to over-fit. Previous studies showed that IH is derived from Bayes’ theorem:

(7) $$P(h|t)=\frac{P(t|h)P(h)}{P(t)}=\frac{\prod_{i=1}^{\left|t\right|}P(x_{i},y_{i}|h)P(h)}{P(t)}=\frac{\prod_{i=1}^{\left|t\right|}P(y_{i}|x_{i},h)P(x_{i}|h)P(h)}{P(t)}$$

where h represents a function that maps input features to their associated tags, t is the training data, $p(y_{i}|x_{i},h)$ denotes the probability that the mapping function uses the mark $y_{i}$ as the label of the input eigenvector $x_{i}$. The larger the $p(y_{i}|x_{i},h)$ is, the greater the probability that the correct label will be assigned to $x_{i}$. The instance hardness of data point ($x_{i},y_{i}$) can be obtained as:

(8) $$IH(<x_{i},y_{i}>)=1-p(y_{i}|x_{i},h)$$

The instance hardness threshold (IHT), a downsampling method, can be developed based on this methodology. In IHT, the dataset can be re-balanced by removing the data points with higher IH values in most classes. This data re-balance process requires an estimator to seek a good performance while applying thresholds to remove data points. There are many types of estimators, including random forests [43], decision trees [44], Adaboost, etc. In this work, a logistic regression as estimator, which was proved with higher efficiency and can balance the data set to 1:1 [45]. After the IHT sampling method, the number of negative samples in the data set changed from 8158 to 2297, the same number as the positive samples.

#### 4.3.2. Repeated Edited Nearest Neighbors

The repeated edited nearest neighbors (RENN) algorithm is another downsampling method, which can be used to re-balance the original dataset by removing the noise points, and it can be implemented as Algorithm 1. After the RENN [46] sampling, the number of positive samples within the dataset becomes 2131, and the number of negative samples changes from 8158 to 2297.
**Algorithm 1.** RENN algorithm.**Input:** The original data set D.             $X_{i}$ is the sample in D.**For i = 1,2,…,n**a. Calculate the Euclidean distances between $X_{i}$ and other samples in D. b. Get the category information of three samples closest to $X_{i}$. c. If two or more nearest samples’ labels are different from $X_{i}$, $X_{i}$ is removed from D. Repeat the above step until $X_{i}$ cannot be removed. **END****Output:** The balanced data set $D_{S}$.

### 4.4. XGBoost Algorithm

The basic idea of XGBoost is that for a given training set $D{=\{(x}_{n}{,y}_{n}{)\}}_{n=1}^{N}$, the k classifications or regression tree sets trained $F=\{f_{1}(x),f_{2}(x),\ldots ,f_{k}(x)\}$ will assign each output sample to different leaf nodes according to the division points of the attribute values, and each leaf node corresponds to one The real-time score $f_{k}$, when given the sample $x_{i}$ that needs to be predicted, the prediction result for that sample is the sum of the prediction results of each tree. The specific model is as follows:

(9) $${\hat{y}}_{i}=\sum_{k=1}^{K}f_{k}(x_{i}),f_{k}\in F$$

where F is the space of all classification trees and regression trees, ${\hat{y}}_{i}$ corresponds to the prediction result of $x_{i}$, $f_{k}(x_{i})$ represents the prediction score of leaf nodes obtained after input of sample $x_{i}$ into kth trees.

The objective function Obj(θ) of the model can be defined as:

(10) $$Obj(\theta )=\sum_{i=1}^{n}l(y_{i},{\hat{y}}_{i})+\sum_{k=1}^{K}\Omega (f_{k})$$

where θ is the parameters of the model. It can be seen that the optimization goal of the model mainly consists of two parts. The first part $\sum_{i=1}^{n}l(y_{i},{\hat{y}}_{i})$ refers to the error function of the model, where the l(x,y) function is the defined error function. The second part $\sum_{k=1}^{K}\Omega (f_{k})$ is the regularization term of the model, indicating the complexity of the k tree.

### 4.5. Protein Interaction Sites Prediction

**Figure 5:**
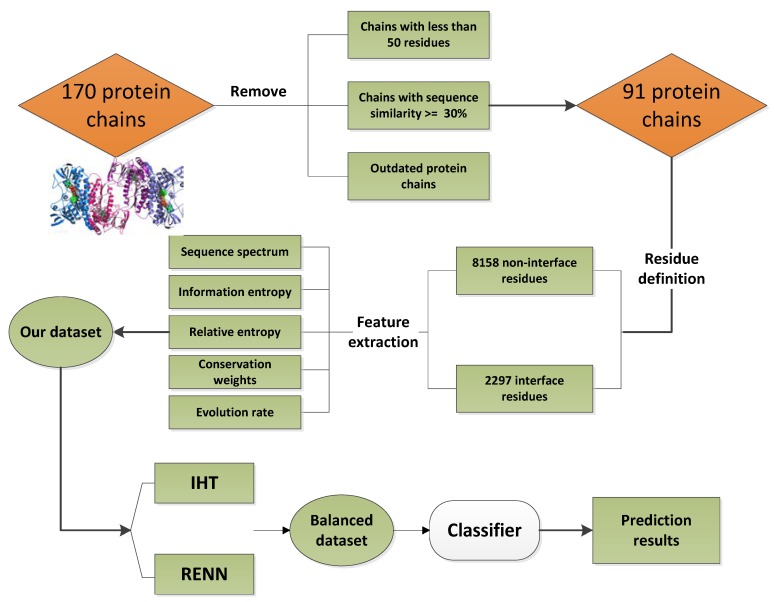
The flowchart of our method.

In this work, each surface residue is represented by the feature coding scheme as a 264-dimensional vector, and the original dataset is processed by the two kinds of downsampling algorithms, i.e., RENN and IHT re-balance the number of positives and negatives. Based on the re-balanced dataset, XGBoost is adopted for protein interaction sites prediction. Herein, to ensure the reliability and stability of the prediction results, a 10-fold cross-validation strategy has been used for prediction model construction. In one round of cross-validation, the original dataset is partitioned into 10 subsets, the first one of them is selected as test set, and the other nine as training set, and then the second subset is selected test set, and other nine as a training set, and so on. In one round of cross-validation, each of 10 subsets is used as test set one time, and overall result is adopted to evaluate the predictions. The flowchart of our proposed method can be found in Figure 5.

## 5. Conclusions

This paper presents a method, XGBoost, for predicting protein-protein interaction sites based on unbalanced data processing strategies. Ninety-one data chains were first obtained through a series of processing in the dataset, and 10,455 surface residues were used for the predictor construction. There are 2297 interface residues and 8158 non-interface residues in the original dataset, which is obviously imbalanced. Based on a hypothesis that there is a large number of overlapping data regions in the dataset, two unbalanced data sampling methods, i.e., RENN and IHT, were proposed for processing data overlap regions, and obtain good prediction performance in the classifier. Among them, IHT-XGB can achieve better prediction performance, such as 80.71% of accuracy rate, 0.614 of MCC. This work shows that the imbalance treatment strategy can improve the prediction of protein-protein interaction sites, and the prediction results is of great significance for understanding life activities and cell activity.

## Figures and Tables

**Table 1:** Sample numbers within the original and re-balance datasets.

	Samples	
	Positive	Negative
Original data	2297	8158
RENN	2297	2131
IHT	2297	2297

**Table 2:** The prediction performance of two re-balance models.

	Acc	F-measure	Pre	Spe	Sen	MCC
RENN-XGB	0.707	0.731	0.699	0.645	0.765	0.454
IHT-XGB	0.807	0.808	0.804	0.802	0.812	0.614

**Table 3:** Classification results of two sampling methods and imbalance dataset.

	Sample		Results			
	Positive	Negative	TP	TN	FP	FN
Imbalanced data	2297	8158	5	8151	25	2249
RENN	2131	2297	1758	1376	755	539
IHT	2291	2297	1864	1844	454	432

**Table 4:** Prediction performance in benchmark datasets.

	Method	Acc	F-measure	Pre	Spe	Sen	MCC
Dset_186	SSWRF	0.679	0.386	0.322	0.697	0.581	0.234
	LORIS	0.604	0.384	0.287	0.586	**0.698**	0.221
	PSIVER	0.673	0.353	0.306	0.743	0.416	0.151
	SCRIBER	0.78	0.279	0.279	0.87	0.279	0.15
	DELPHI	**0.803**	0.353	0.353	**0.884**	0.352	0.235
	IHT_XGB	0.716	**0.694**	**0.753**	0.788	0.644	**0.437**
Dset_72	SSWRF	0.648	0.351	0.267	0.643	0.654	0.224
	LORIS	0.614	0.324	0.238	0.610	0.631	0.177
	PSIVER	0.661	0.278	0.25	0.693	0.465	0.135
	SCRIBER	0.837	0.232	0.232	0.909	0.232	0.141
	DELPHI	**0.847**	0.275	0.276	**0.915**	0.274	0.189
	IHT_XGB	0.702	**0.689**	**0.721**	0.741	**0.663**	**0.405**
Dset_164	SSWRF	0.621	0.365	0.323	0.656	0.527	0.152
	LORIS	0.588	0.323	0.263	0.609	0.538	0.111
	PSIVER	0.596	0.295	0.253	0.634	0.464	0.078
	SCRIBER	0.756	0.327	0.327	0.851	0.327	0.179
	DELPHI	**0.758**	0.332	0.332	**0.852**	0.332	0.184
	IHT_XGB	0.733	**0.715**	**0.767**	0.795	**0.671**	**0.470**
